# Hair Proteome Variation at Different Body Locations on Genetically Variant Peptide Detection for Protein-Based Human Identification

**DOI:** 10.1038/s41598-019-44007-7

**Published:** 2019-05-21

**Authors:** Fanny Chu, Katelyn E. Mason, Deon S. Anex, A. Daniel Jones, Bradley R. Hart

**Affiliations:** 1 Forensic Science Center, Lawrence Livermore National Laboratory, 7000 East Ave., Livermore, CA 94550 USA; 2 Department of Chemistry, Michigan State University, 578 S Shaw Ln, East Lansing, MI 48824 USA; 3 Department of Biochemistry and Molecular Biology, Michigan State University, 603 Wilson Rd, East Lansing, MI 48824 USA

**Keywords:** Proteomics, Genetic markers

## Abstract

Human hair contains minimal intact nuclear DNA for human identification in forensic and archaeological applications. In contrast, proteins offer a pathway to exploit hair evidence for human identification owing to their persistence, abundance, and derivation from DNA. Individualizing single nucleotide polymorphisms (SNPs) are often conserved as single amino acid polymorphisms in genetically variant peptides (GVPs). Detection of GVP markers in the hair proteome via high-resolution tandem mass spectrometry permits inference of SNPs with known statistical probabilities. To adopt this approach for forensic investigations, hair proteomic variation and its effects on GVP identification must first be characterized. This research aimed to assess variation in single-inch head, arm, and pubic hair, and discover body location-invariant GVP markers to distinguish individuals. Comparison of protein profiles revealed greater body location-specific variation in keratin-associated proteins and intracellular proteins, allowing body location differentiation. However, robust GVP markers derive primarily from keratins that do not exhibit body location-specific differential expression, supporting GVP identification independence from hair proteomic variation at the various body locations. Further, pairwise comparisons of GVP profiles with 8 SNPs demonstrated greatest interindividual variation and high intraindividual consistency, enabling similar differentiative potential of individuals using single hairs irrespective of body location origin.

## Introduction

Human hair, as one of the few biological specimen types that persist for long periods of time, is invaluable to forensic and archaeological investigations, yet limited identification information is often obtained from hair using conventional approaches. Comprised primarily of keratins and keratin-associated proteins, hair exhibits high durability that contributes to its persistence. Packed into coiled-coils and localized to the cortex, cuticle, or medulla of the hair shaft^1,2^, hair keratins are stabilized and provide tensile strength via cross-linking by cystine disulfide bonds^3^. Differences in their amino acid composition further separate them into type I acidic keratins (K31, K32, K33A, K33B, K34 – K40) with low-sulfur content and type II neutral to basic keratins (K81 – K86) with high-sulfur content^2–6^. Keratin-associated proteins (KAPs), categorized by their amino acid composition as high-sulfur, ultra-high sulfur, or high glycine-tyrosine proteins, participate in cross-linking alongside keratins to provide rigidity to the matrix^7,8^.

In contrast to its high protein content, hair contains minimal intact nuclear DNA, which is the preferred evidence type for human identification in forensic investigations owing to its specificity for individualization and low associated statistical probabilities. Found within keratinized corneocytes, nucleases including DNase1L2 degrade DNA to tiny fragments, depending on their abundances and catalytic activities^9^. This process begins immediately as the keratinocyte moves out of the hair follicle and terminal differentiation occurs^10^, and exhibits high interindividual variation^9^. With continued hair growth, DNA degrades to low and variable amounts, making forensic nuclear DNA profiling in hair unreliable. When complete analysis of nuclear DNA, the gold standard, either through short tandem repeat profiling^10–12^ or with single nucleotide polymorphisms (SNPs)^13–15^, is not possible, an alternative method for human identification becomes critically important.

Proteins offer an attractive pathway for identification, as they are robust and derive from DNA. Protein-based identification methods are largely supported by advancements in proteomics, such as faster-scanning high-resolution mass analyzers and bioinformatics tools. The proteome is a focus for biomarker identification indicative of various disease states and also for analysis of ancient specimens, since minimal DNA remains owing to the ubiquitous nature of nucleases in the environment^16,17^, but proteins are likely to be preserved. High-resolution mass spectrometry analysis of fossil bones revealed survival of proteins in the leucine-rich repeat family and serum proteins in samples dating back to 900 thousand years^18^. Peptide markers characterized in various keratinous tissues of agricultural and archaeological importance, including horn and baleen, enabled species identification within the mammalian kingdom based on variant amino acid sequences that distinguish genera^19^. Proteomics methods for biological fluid identification have also identified peptide markers specific to fluids including human saliva, urine, seminal fluid, and vaginal fluid, demonstrating the potential of protein detection and identification for utility in forensic investigations^20,21^. While the hair proteome has been probed to characterize disorders including lamellar ichthyosis and trichothiodystrophy using protein expression differences^8,22,23^, hair proteins have received little attention for human identification.

The few reports of distinguishing individuals from hair proteomes include a 2014 paper by Laatsch and colleagues who analyzed protein composition and expression differences in hair from different ethnic populations and from various body locations and found specific protein expression differences to differentiate based upon ethnicity and body location^24^. Following in this vein, Wu *et al*. showed in 2017 that protein profiles differentiated monozygotic twins from unrelated individuals^25^. However, both studies focused on protein abundances to provide differentiation of different populations, which does not provide sufficient specificity to distinguish individuals, as hair protein levels are evolutionarily conserved within ethnic populations.

Alternatively, genetically variant peptides (GVPs) in hair proteins possess great potential for differentiation of individuals. Identification of single amino acid polymorphisms in GVPs permits inference of individualizing SNPs. Parker *et al*. first demonstrated the identification and use of single amino acid polymorphisms in GVPs from head hair to differentiate individuals^26^. Further, analogous to a SNP panel developed by Pakstis *et al*.^14^, Parker and co-workers compiled a panel of 33 SNPs identified from GVP markers and verified by Sanger sequencing, and determined random match probabilities ranging up to 1 in 14,000 for a cohort of 60 subjects^26^. Mason *et al*. then showed comparable performance in single one-inch hair analysis^27^ to that using bulk quantities of scalp hair in Parker *et al*.^26^, demonstrating the feasibility of protein-based human identification for forensic investigations. Although our protein-based identification approach has been adapted to simulate forensically-relevant sample sizes via single hair analysis, fundamental questions regarding hair protein chemistry and its effects on GVP marker identification remain.

One key knowledge gap lies in whether the same GVP markers can be identified in hair from different body locations, as hair origin is often not known from hair specimens recovered for forensic investigations. From work performed by Laatsch *et al*., differential protein expression in hair from a few body locations has been characterized^24^. However, effects of body location-specific protein abundance variation on robust identification of GVP markers have not yet been elucidated. We employ proteomics technologies and methodologies developed for single hair analysis in this study to examine GVP markers identified from head, arm, and pubic hair for any differences in the differentiative potential of individuals. Aims of this research include the determination of body location-specific proteomic variation, evaluation of the effects of differential protein expression on GVP identification and subsequent SNP inference, and quantification of the extent to which individuals are differentiated with robust, i.e., consistently identified and body location-invariant, GVP markers. We demonstrate the independence of forensic SNP identification from body location-specific hair proteomic variation and further identify viable GVP markers that yield similar distinction of individuals irrespective of body location origin in single one-inch (25 mm) hairs.

## Results

### Single inch hair sample preparation performance

Single one-inch hairs yield rich protein profiles that are comparable to profiles established with greater hair quantities; on average, 142 ± 33 (s.d.) proteins were identified from each of 9 head hairs (i.e., from three sets of proteomics-only biological replicates from three individuals), and the average number of identified unique peptides was 1,031 ± 219. From unique peptides, the average numbers of identified amino acids were 15,527 ± 3,056. The presence of a subset of unique peptides known as genetically variant peptides (GVPs) enabled inference of 16 ± 5 SNPs from major GVPs, and 17 ± 3 SNPs from minor GVPs (i.e., GVPs corresponding to the major and minor alleles, respectively). Because both major or minor GVPs allow SNP inference, non-synonymous, or missense, SNPs were reported for both types of GVPs. However, in some cases, detection of both GVPs for the same SNP may not be possible. In previous studies, Parker *et al*. identified at least 180 proteins in 10 mg of head hair samples from 60 subjects and detected between 156 and 2,011 unique peptides^26^, and Adav *et al*. identified, on average, 195 ± 12 proteins in human hair using various sample preparation methods^28^. Commensurate performance to previous works is achieved even when sample size is substantially reduced to simulate amounts of material available from forensic samples.

In addition, performing co-extraction of protein and mitochondrial DNA yielded no loss in protein information relative to processing for protein alone. Proteomic results from co-extraction were not statistically different from proteomics-only sample preparation for each of the above metrics (two sample t-test; p ≥ 0.106; Supplementary Fig. S1); for example, 156 ± 56 proteins were identified from proteomics-only samples and 151 ± 39 proteins were detected in co-extracted samples. These observations indicate that additional steps taken to co-extract DNA with protein did not adversely affect protein identification or detection of unique peptides and missense SNPs from GVPs. As both sample preparation methods yielded the same proteomic information, the protein/DNA co-extracted sample set was included in this study for all further analyses. Analysis of GVPs and mtDNA can provide corroborating evidence for more confident profiling of individuals, which will be explored in a later publication.

### Proteomic variation at different body locations

**Figure 1:**
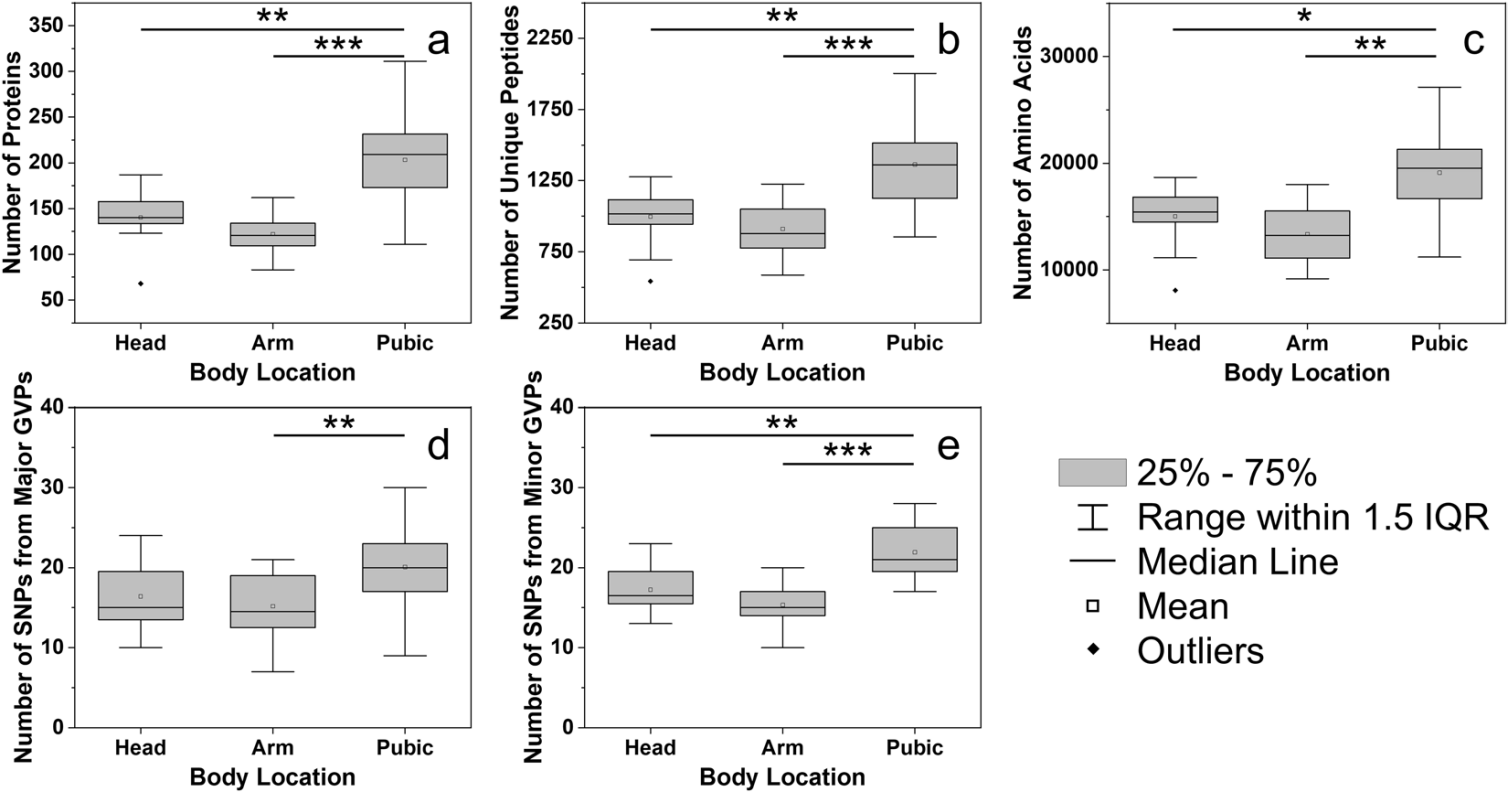
Comparison of numbers of identified (**a**) proteins, (**b**) unique peptides, (**c**) amino acids, and missense SNPs inferred from (**d**) major and (**e**) minor GVPs at different body locations. Black lines represent statistically significant comparisons and significance levels are represented as: p ≤ 0.05 (*), p ≤ 0.01 (**), and p ≤ 0.001 (***). Pubic hair samples yield statistically greater numbers of proteins, peptides, amino acids, and inferred SNPs (two-way ANOVA and Tukey HSD; n = 36).

Hair proteomic variation at three different body locations in 36 hair specimens was first assessed by comparing five metrics: the numbers of detected proteins, unique peptides, amino acids, and missense SNPs from major and minor GVPs (Fig. 1). Two-way ANOVAs with Tukey HSD post-hoc tests were performed for each metric to account for effects of body location and individual. Statistical testing revealed significant effects of body location on the numbers of detected proteins (p = 1.07 × 10^−4^), unique peptides (p = 5.66 × 10^−4^), and amino acids (p = 2.21 × 10^−3^), while effects of individual and the interaction between body location and individual were not significant. A single inch of pubic hair yields more proteins, unique peptides, and amino acids, than head or arm hair. A significant effect of body location on the number of SNPs inferred from GVPs was observed for major (p = 7.56 × 10^−3^) and minor GVPs (p = 1.91 × 10^−5^). These results suggest that compared to head and arm hair, the protein composition of pubic hair is more complex, from which many GVPs and SNPs can be identified for human identification.

Significant effects of body location observed for these five metrics may arise from differences in mass per unit length of hair. The mass of a single inch of pubic hair (200.1 ± 39.6 µg) was statistically greater than an inch of head (84.4 ± 27.7 µg; two-way ANOVA and Tukey HSD; p = 1.76 × 10^−9^) or arm hair (49.4 ± 22.2 µg; p = 1.74 × 10^−11^). Despite mass differences in hair, the same injection volume was used for each sample, and thus, different quantities of material were loaded onto the column for LC-MS/MS. It is proposed that more proteins, unique peptides, amino acids, and inferred SNPs were identified in pubic hair samples owing to larger on-column mass loadings.

To assess body location-specific proteomic variation without bias from different on-column mass loadings, protein abundances were examined after normalization to total chromatographic peak area of identified peptides. A previous study by Laatsch and co-workers reported differential expression at different body locations for a subset of proteins^24^. To confirm these observations in this study for head and pubic hair, and to assess differential protein expression in arm hair, which was not examined previously, protein quantities derived from mass spectral data were compared. Various approaches have been utilized to quantify proteins using mass spectral data, including spectral counts^24,29,30^, precursor ion peak areas from MS scans^31,32^, and MS/MS fragment ion abundances^33^ to represent peptide abundance. Because dynamic exclusion was used during data acquisition to maximize peptide identification and protein coverage, MS/MS spectral counts do not reliably represent peptide abundance, especially lower abundance peptides^34^. We chose to use the more robust of the latter two methods and tabulated MS scan precursor ion peak areas in mass spectral data from a complete list of identified unique peptides. Bias towards samples with larger mass loadings was removed by normalizing each precursor ion peak area to the total peak area of all identified peptides. Protein abundance in each sample was calculated as the sum of all normalized peak areas assigned to the protein.

**Figure 2:**
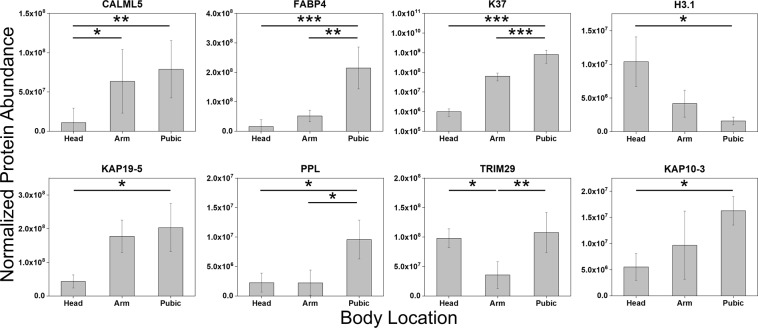
Average abundances for a subset of differentially expressed hair proteins at different body locations (two-way ANOVA and Tukey HSD; n = 36). Error bars represent standard deviation from 4 replicate measurements of each of three individuals. Black lines represent statistically significant comparisons and significance levels are represented as: p ≤ 0.05 (*), p ≤ 0.01 (**), and p ≤ 0.001 (***).

Protein abundance was examined in this study to observe any effects of body location. Statistical comparison of protein abundances identified 37 proteins with body location-specific differential expression, of which a subset is shown in Fig. 2 (two-way ANOVA and Tukey HSD). Further, many differentially expressed proteins show higher expression in pubic hair and are least abundant in arm hair, suggesting that pubic hair not only comprises a complex set of proteins, but also that proteins are more abundant in pubic hair compared to head and arm hair, even after accounting for mass differences. Not surprisingly, keratins and KAPs comprise only 27% of body location-specific differentially expressed proteins (i.e., 10 proteins), while intracellular proteins such as FABP4, MIF, and ATP5B make up the majority. As keratins and KAPs primarily contribute to the structural integrity of hair, which is highly conserved, it is unlikely that many hair structural proteins would exhibit differential expression at the various body locations. Many intracellular proteins are also least abundant in arm hair, although arm hair samples have notably high abundances of CALML5, GSDMA, and KAP19-5 compared to head hair samples. While the protein abundance profiles of head and arm hair samples are more similar compared to pubic hair, protein abundance variation in 37 markers enabled distinction of hair fibers from different body locations via principal components analysis (Supplementary Fig. S2). Differential protein expression captured with protein abundance confirms proteomic variation in hair from different body locations.

### Effects of proteomic variation on GVP identification

Because protein abundances vary for a subset of hair proteins at different body locations and GVPs result from hair protein digests, it was considered that GVP identification may be affected by body location-specific differential protein expression. Therefore, it was imperative to examine the SNPs identified in each sample and determine whether differential protein expression affects GVP identification and subsequent SNP inference. Further comparison of identified SNPs in each sample was performed to observe whether some SNPs are only identified at specific body locations. Only SNP inferences consistent with an individual’s genotype determined from exome sequencing were considered. SNPs with false positive responses are not robust candidates for a GVP panel and were removed; 65 SNPs remained for further analysis.

**Figure 3:**
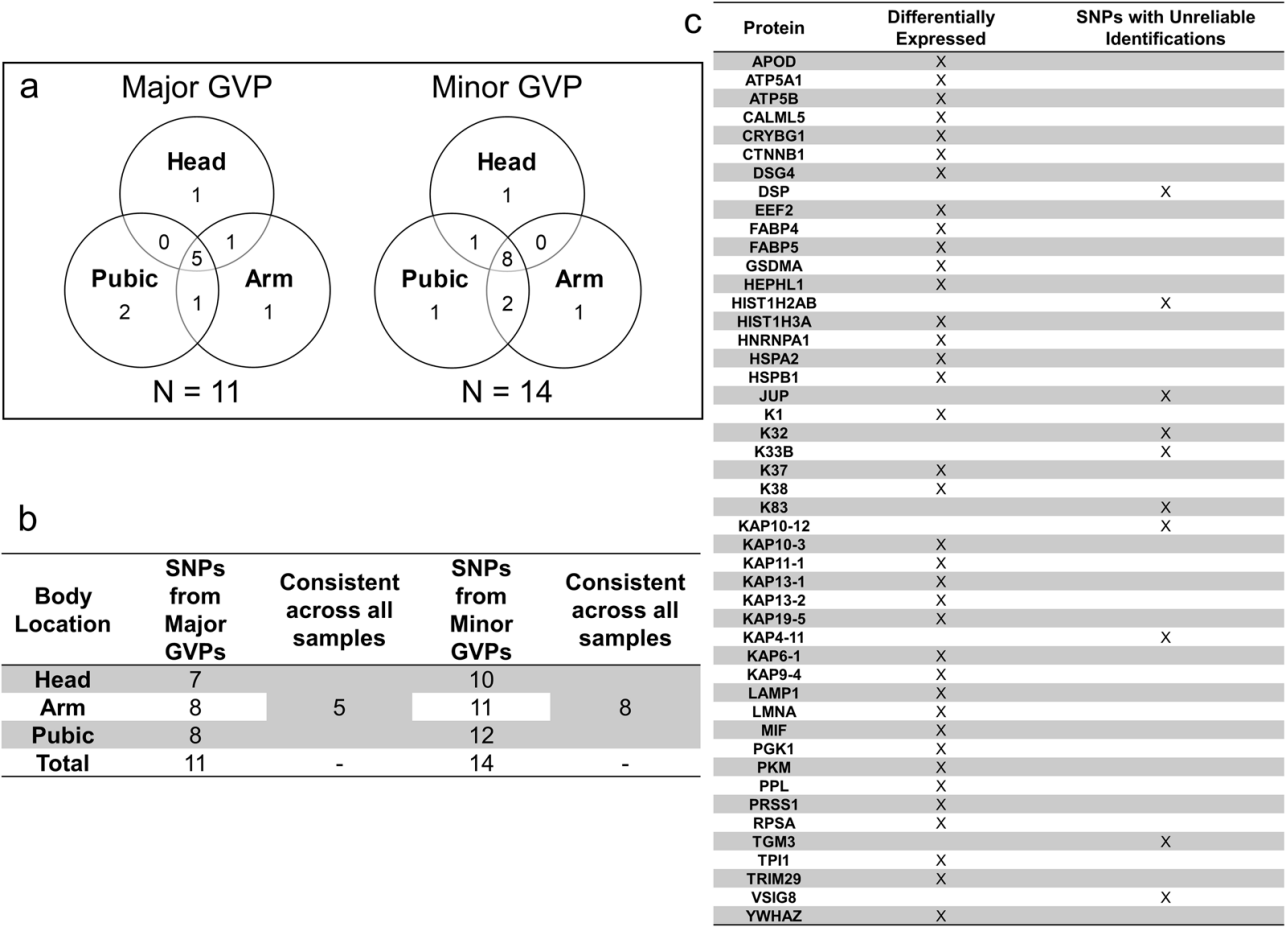
Comparison and distribution of exome-proteome consistent SNPs across different body locations. (**a**) Distribution of inferred consistent SNPs across the three body locations for major and minor GVPs, respectively. (**b**) Summary of the number of consistent SNPs at each body location. (**c**) Comparison of differentially expressed proteins to proteins of 11 SNPs with unreliable identifications at one or two body locations (i.e., not identified at all body locations). The majority of exome-proteome consistent SNPs identified at each body location are identified in all samples. Unreliably identified SNPs at either one or two body locations originate from a set of proteins that are not differentially expressed; there is no overlap between these sets of proteins. Therefore, SNPs are not body location-specific.

To observe any localization of SNPs, distributions of inferred SNPs from major and minor GVPs were compared across body locations. Of 65 SNPs, only exome-proteome consistent SNPs, in which the proteomic response corresponded with the exome response, i.e., true positive and true negative responses, across all 12 samples per body location for either major or minor GVPs, were retained (Fig. 3). Figure 3a,b illustrate the amount of overlap in consistent SNPs across samples from different body locations. From 11 and 14 consistent SNPs identified from major and minor GVPs, respectively, 5 and 8 SNPs are identified at all body locations, which comprise the majority (on average, 69%) of exome-proteome consistent SNPs. This observation suggests that reliable SNP identification in samples within a body location often extends to all samples. Only 11 SNPs in total are not identified at all body locations; there is one unreliably identified SNP that overlaps between major and minor GVPs.

The possibility that body location-specific SNP localization results from proteomic variation was further examined by comparing subsets of proteins. The subset of 37 proteins with body location-specific differential expression was compared with the proteins of 11 inconsistently identified SNPs (Fig. 3c). Any overlap in composition would indicate that differential expression of the protein affects downstream GVP identification and SNP inference within that protein. However, no overlap existed between differentially expressed proteins and proteins containing unreliably identified SNPs. With the exception of five proteins (APOD, CALML5, GSDMA, K37, KAP10-3), SNPs are not identified in body location-specific differentially expressed proteins. Despite significant positive correlations between the frequency of identifying SNPs from 3 of these proteins and protein abundance (Pearson product-moment correlation; p ≤ 0.043; Supplementary Fig. S3), identification of these SNPs remains variable among sample replicates, regardless of body location. Further, no statistical positive correlation between SNP identification frequency and protein abundance was found for unreliably identified SNPs (Supplementary Fig. S3), demonstrating that body location-specific differential expression is not linked to SNP identification for all exome-proteome consistent SNPs. Therefore, while expression of APOD, GSDMA, and K37 may display some correlation with SNP identification, the vast majority (on average, 97%) of GVP identification is not affected by differential protein expression, especially if the peptides are consistently identified among sample replicates. SNP identification in hair specimens is not dependent on body location. GVP identification from protein digests of hair specimens is equally viable regardless of body location origin and all detected GVPs are candidates for a GVP panel.

### GVP candidates for human identification panel

A series of criteria were established to evaluate GVP candidates for a robust panel. First, only GVPs that indicate exome-proteome consistent SNPs were considered. Furthermore, only consistent SNPs identified in all samples were selected, as these SNPs have the lowest false negative rates and their GVP counterparts have the highest chance of being detected. After accounting for overlap between major and minor GVPs, 12 SNPs remained for consideration. SNP identifiers, the two most abundant forms of the GVP, and their MS scan precursor ion abundances are reported in Table 1. See Supplementary Table S1 for a complete list of GVPs.

**Table 1:** SNP and GVP candidates for GVP panel.

Gene	SNP Identifier	Amino Acid Polymorphism	GVP Type	Peptide^†^	PTM	Average Abundance	Observation Frequency
FAM83H	rs9969600	Q/H	Minor	R.VNL**H**HVDFLR		1.10 × 10^6^	2
KRT32	rs2071563	T/M	Major	R.ARLEGEIN**T**YR	A1:Formylation	5.72 × 10^7^	27
KRT32	rs2071563	T/M	Major	R.LEGEIN**T**YR		1.71 × 10^8^	28
KRT32	rs2071563	T/M	Minor	R.ARLEGEIN**M**YR	M9:Oxidation (M)	4.79 × 10^7^	10
KRT32	rs2071563	T/M	Minor	R.LEGEIN**M**YR	M7:Oxidation (M)	1.69 × 10^7^	7
KRT33A	rs148752041	D/H	Minor	R.**H**NAELENLIR	N2:Deamidation (NQ)	4.38 × 10^7^	8
KRT33A	rs148752041	D/H	Minor	R.**H**NAELENLIRER		1.11 × 10^8^	7
KRT33B	17:g.41366553 G > T *	L/M	Major	R.ILDE**L**TLCRSDLEAQMESLKEELLSLKQNHEQEVNTLR	C8:Carbamidomethylation;N29:Deamidation (NQ)	9.11 × 10^7^	12
KRT33B	17:g.41366553 G > T *	L/M	Major	R.ILDE**L**TLCRSDLEAQMESLKEELLSLK	C8:Carbamidomethylation	1.49 × 10^7^	11
KRT33B	17:g.41366553 G > T *	L/M	Minor	R.ILDE**M**TLCR	C8:Carbamidomethylation	7.10 × 10^7^	10
KRT33B	17:g.41366553 G > T *	L/M	Minor	R.RILDE**M**TLCR	C9:Carbamidomethylation	2.20 × 10^7^	7
KRT81	rs2071588	G/R	Minor	R.GLTGGFGSHSVC**R**	C12:Carbamidomethylation	3.35 × 10^8^	19
KRT81	rs2071588	G/R	Minor	L.TGGFGSHSVC**R**	C10:Carbamidomethylation	1.18 × 10^7^	13
KRT83	rs2852464	I/M	Major	R.DLNMDC**I**VAEIK	C6:Carbamidomethylation	1.08 × 10^8^	32
KRT83	rs2852464	I/M	Major	R.DLNMDC**I**VAEIKAQYDDIATR	K12:Carbamylation	1.83 × 10^8^	23
KRT83	rs2852464	I/M	Minor	R.DLNMDC**M**VAEIK	C6:Carbamidomethylation	2.97 × 10^7^	19
KRT83	rs2852464	I/M	Minor	R.DLNMDC**M**VAEIKAQYDDIATR	C6:Carbamidomethylation;M7:Oxidation (M)	4.29 × 10^6^	16
KRTAP10-3	rs233252	C/Y	Minor	R.ST**Y**CVPIPSC	C4:Carbamidomethylation;C10:Carbamidomethylation	2.97 × 10^6^	4
KRTAP10-3	rs233252	C/Y	Minor	R.ST**Y**CVPIPS	C4:Carbamidomethylation	1.65 × 10^6^	2
KRTAP10-9	rs9980129	R/C	Minor	C.CAPTSS**C**QPSYCR	C1:Carbamidomethylation;C7:Carbamidomethylation; C12:Carbamidomethylation	6.99 × 10^6^	10
KRTAP4-11	rs760092771	S/C	Major	R.TTYCRPSCCVS**S**	C4:Carbamidomethylation;C8:Carbamidomethylation; C9:Carbamidomethylation	1.75 × 10^8^	5
KRTAP4-11	rs760092771	S/C	Minor	R.TTYCRPSYSVS**C**C	C4:Carbamidomethylation;C12:Carbamidomethylation; C13:Carbamidomethylation	1.32 × 10^8^	12
KRTAP4-11	rs760092771	S/C	Minor	R.TTYCRPSYSVS**C**	C4:Carbamidomethylation;C12:Carbamidomethylation	8.10 × 10^7^	11
KRTAP4-11	rs763737606	C/S	Major	R.TTYCRPSC**C**VSS	C4:Carbamidomethylation;C8:Carbamidomethylation; C9:Carbamidomethylation	1.75 × 10^8^	5
KRTAP4-11	rs763737606	C/S	Minor	R.TTYCRPSY**S**VSCC	C4:Carbamidomethylation;C12:Carbamidomethylation; C13:Carbamidomethylation	1.32 × 10^8^	12
KRTAP4-11	rs763737606	C/S	Minor	R.TTYCRPSY**S**VSC	C4:Carbamidomethylation;C12:Carbamidomethylation	8.10 × 10^7^	11
KRTAP4-11	rs774046661	C/Y	Major	R.TTYCRPS**C**CVSS	C4:Carbamidomethylation;C8:Carbamidomethylation; C9:Carbamidomethylation	1.75 × 10^8^	5
KRTAP4-11	rs774046661	C/Y	Minor	R.TTYCRPS**Y**SVSCC	C4:Carbamidomethylation;C12:Carbamidomethylation; C13:Carbamidomethylation	1.32 × 10^8^	12
KRTAP4-11	rs774046661	C/Y	Minor	R.TTYCRPS**Y**SVSC	C4:Carbamidomethylation;C12:Carbamidomethylation	8.10 × 10^7^	11
VSIG8	rs62624468	V/I	Major	R.LGCPY**V**LDPEDYGPNGLDIEWMQVNSDPAHHR	C3:Carbamidomethylation;N15:Deamidation (NQ)	1.36 × 10^7^	18
VSIG8	rs62624468	V/I	Major	R.LGCPY**V**LDPEDYGPNGLDIEWMQVNSDPAHHRENVFLSYQDKR	C3:Carbamidomethylation;N15:Deamidation (NQ);M22:Oxidation (M)	6.88 × 10^6^	14
VSIG8	rs62624468	V/I	Minor	R.LGCPY**I**LDPEDYGPNGLDIEWMQVNSDPAHHR	C3:Carbamidomethylation;N15:Deamidation (NQ);M22:Oxidation (M)	4.92 × 10^6^	5
VSIG8	rs62624468	V/I	Minor	R.LGCPY**I**LDPEDYGPNGLDIEWMQVNSDPAHHRENVFLSYQDKR	C3:Carbamidomethylation;N15:Deamidation (NQ);M22:Oxidation (M)	2.90 × 10^6^	3

^*^No SNP identifier associated with SNP (HGVS notation used); Bold text denotes location of amino acid variant in genetically variant peptide; ^†^Preceding amino acid in peptide sequence denoted by “X.”

The second criterion used to evaluate GVP candidates in Table 1 is marker independence for random match probability (RMP) determination at the SNP level. To assess the performance of a robust panel for forensic identifications in a population, random match probabilities are calculated as the product of genotype frequencies for each SNP locus. However, genotype frequencies for correlated SNPs, i.e., SNPs in linkage disequilibrium^35,36^, may be biased in the population, which violates the assumption of marker independence for RMP calculations. To reduce the effect of possible disequilibria, a conservative one-SNP-per-gene rule was adopted; more sophisticated treatment of linkage disequilibrium will allow for inclusion of more GVPs, and thus, lower RMPs. For multiple SNPs from a gene, the SNP with the lowest minor allele frequency was selected. Finally, SNPs without Reference SNP IDs were also not considered further, as genotype frequencies are not known for these candidates. After applying these criteria, 8 SNPs remained for inclusion in a panel from 245 GVPs.

### GVP profiles and identification performance

**Figure 4:**
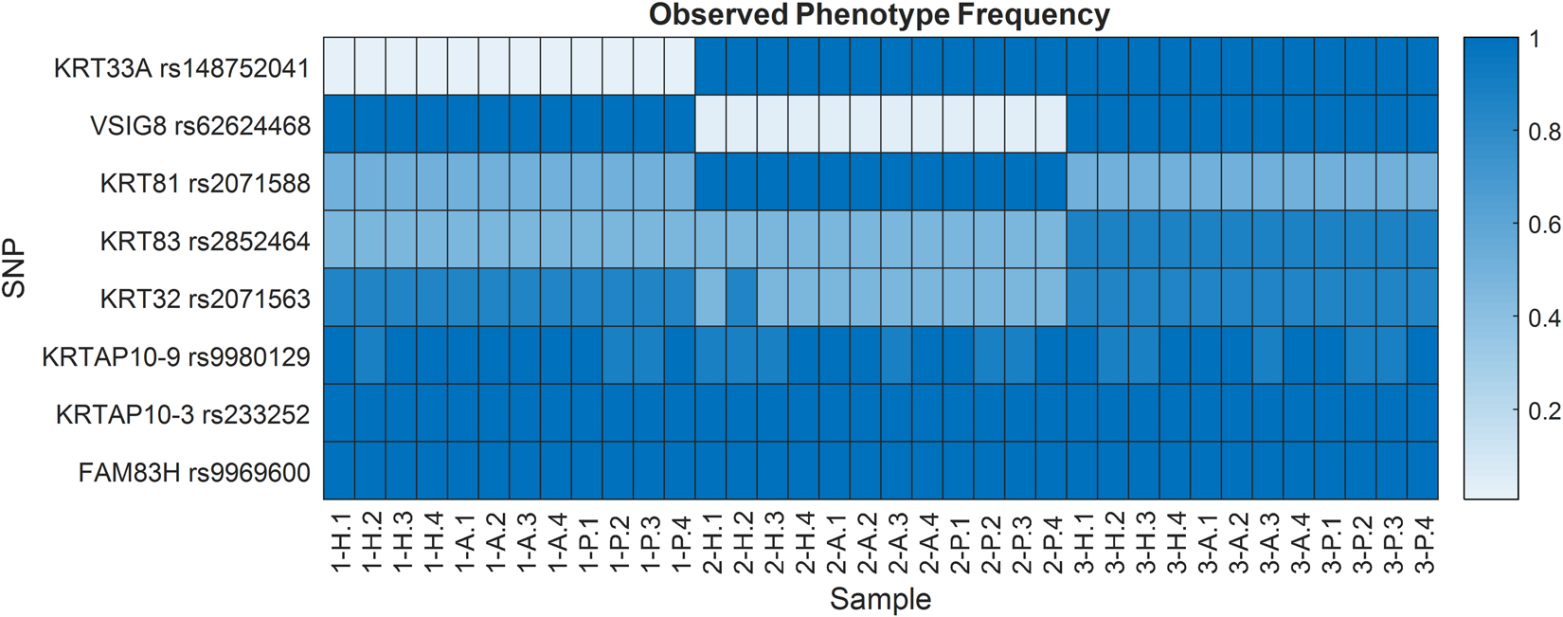
GVP profiles of 36 samples using observed phenotype frequency to represent the presence or absence of major and minor GVPs at 8 SNP loci. Profiles within an individual are similar, indicating consistent identification of SNPs with robust GVPs.

GVP profiles for each sample were established using 8 robust SNPs. Each GVP profile was established using the presence or absence of the major and minor GVPs at each SNP locus. Figure 4 displays a simplified version of each profile by using observed phenotype frequencies to represent the presence or absence of GVPs, as described in Materials and Methods. The full set of profiles that denotes the presence or absence of GVPs is found in Supplementary Fig. S4.

**Figure 5:**
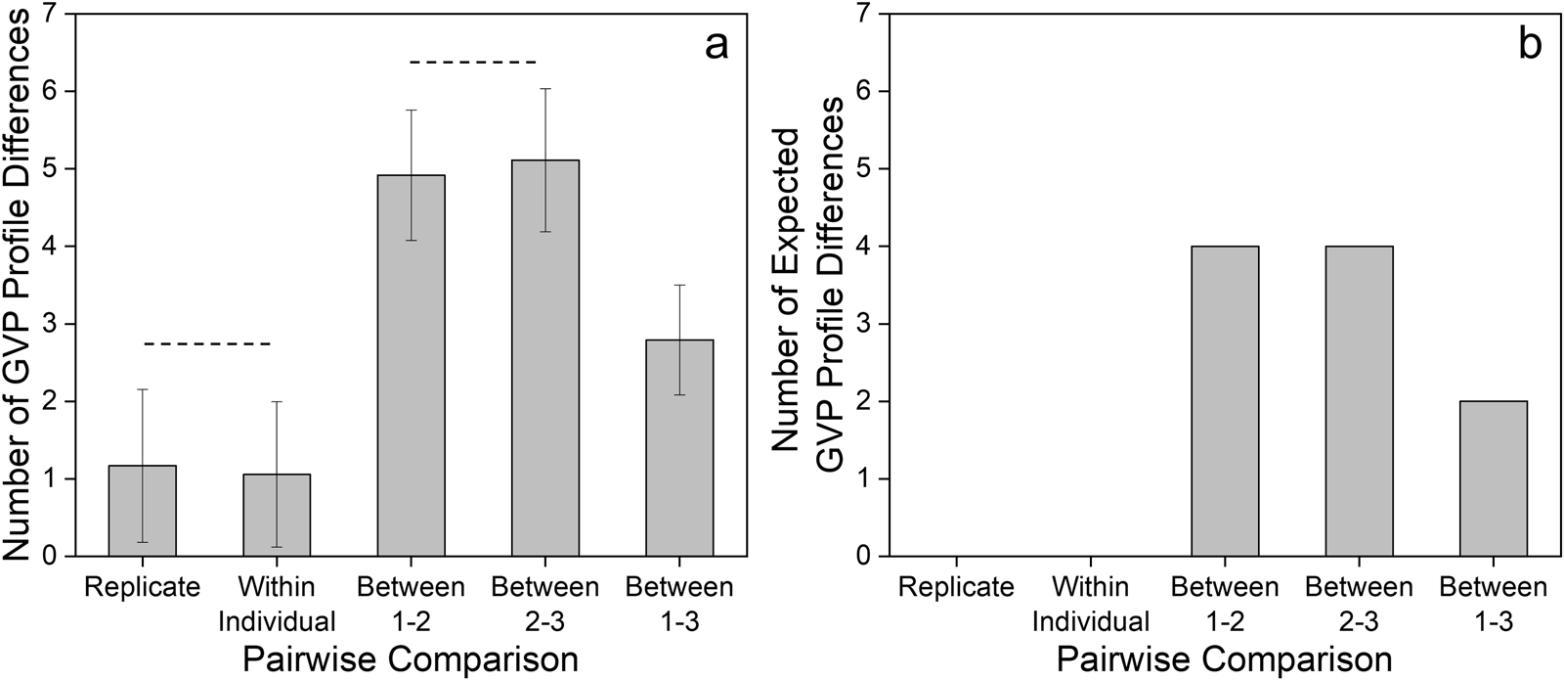
(**a**) Average number of GVP profile differences from different pairwise comparison categories compared to (**b**) expected number of GVP profile differences. Error bars represent the standard deviation. All but two comparisons, denoted by dotted line, are statistically significant (Kruskal-Wallis and Dunn tests; n = 630; p ≤ 3.80 × 10^−6^). The numbers of observed profile differences approximate expected GVP profile differences. Between Individual profile differences are statistically greater than Replicate and Within Individual profile differences.

GVP profiles within an individual, irrespective of body location, are more similar compared to GVP profiles between individuals. Pairwise comparisons of GVP profiles allowed quantification of profile similarity, using the number of observed phenotype differences across 8 SNP loci, termed GVP profile differences. Differences were recorded if the compared responses did not match exactly, and then summed for each pairwise comparison, totaling 630 comparisons. Replicate comparisons, performed between hair specimens from the same individual and body location, yielded 1.17 ± 0.99 GVP profile differences, and within-individual comparisons, between hair samples from the same individual but different body locations, showed 1.06 ± 0.94 differences. As expected, between-individual comparisons exhibited the greatest number of GVP profile differences, with 4.92 ± 0.84, 5.11 ± 0.92, and 2.79 ± 0.71 differences, respectively, between Individuals 1–2, 2–3, and 1–3 (Fig. 5a). All observed profile differences approximate expected GVP profile differences (Fig. 5b). Greatest profile variation lies between individuals (Kruskal-Wallis test; p = 2.96 × 10^−108^), demonstrating that despite some sample replicate and within-individual variation (e.g., body location), distinct GVP profiles are observed in samples from different individuals.

**Figure 6:**
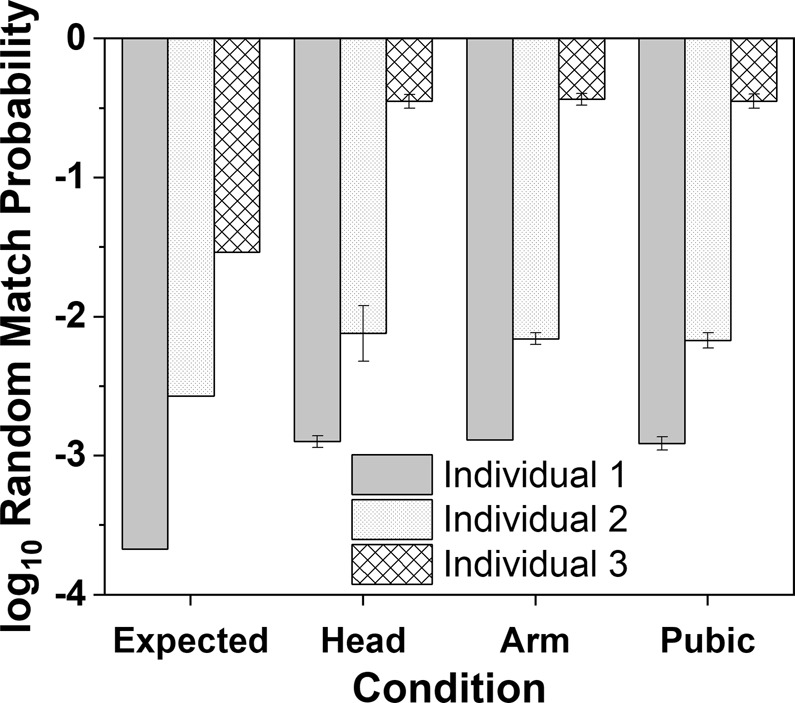
Experimentally observed random match probabilities (m ± 95% CI) compared to expected RMP values for each individual. Expected RMPs are theoretically-derived values based on the detection of all GVPs consistent with an individual’s genotype for the same 8 SNPs. RMP values of different body location samples from the same individual are not different; the extent to which individuals are distinguished from one another is not affected by hair origin. Observed RMP values from a robust set of SNPs approximate expected values within an order of magnitude.

Furthermore, RMPs, derived as products of observed phenotype frequencies from GVP profiles of each sample, align with the individual (Fig. 6). Experimental RMPs range between 1 in 3 and 1 in 870, within an order of magnitude of expected RMPs for each individual. Most importantly, GVP profiles of samples belonging to the same individual enable distinction of the individual to the same extent, regardless of body location, demonstrating that with a robust panel of inferred SNPs from GVPs, the probative value of one-inch head, arm, and pubic hair samples is equivalent within an individual.

## Discussion

This study sought to examine body location-specific proteomic variation and its effects on SNP identification, and quantify the extent to which individuals are distinguished using single one-inch hairs from different body locations.

Many more proteins exhibited higher abundances in pubic hair compared to head and arm hair, highlighting the rich protein profile that can be obtained with pubic hair. Laatsch *et al*. reported differential expression levels for 12 proteins between head and pubic hair using spectral counts^24^. Of these, 6 proteins had statistically different abundances between head and pubic hair samples in the current study, indicating agreement between studies using different protein quantification methods. In particular, K37 was found to be differentially expressed between head and pubic hair and is also statistically more abundant in pubic hair compared to arm hair. The presence of this protein in head hair, albeit at low levels, agrees with findings by previous studies^24,26,28^. We observed unique peptides with a considerable abundance from this protein in only 25% of head hairs, while K37 was consistently expressed in arm and pubic hair. Because the hair samples prior to segmentation were of similar length (1–2 inches), absence of K37 in many head hairs cannot be attributed to degradation with age of hair, i.e., time since biosynthesis. Instead, K37 expression is linked to hair follicle keratinocyte differentiation differences at the various body locations; the protein is known to be expressed in the medulla of sexual hairs, including pubic hair, matured from unmedullated vellus hairs^37^.

Hair structural proteins make up only 27% of differentially expressed proteins (i.e., 10 proteins), in agreement with Laatsch *et al*.^24^. Variation in KAP abundance is more prevalent compared to keratins or other peripheral structural proteins. For example, KAP19-5 is highly abundant in arm and pubic hair compared to head hair, and pubic hair is significantly enriched with many other KAPs, including KAP11-1 and KAP10-3 (two-way ANOVA and Tukey HSD; Supplementary Fig. S5). Differential expression of KAPs may be linked to the structural conformation of intermediate filaments and affect physicochemical properties (e.g., rigidity, tensile strength, thickness) of hair fibers, and can serve as useful markers to differentiate hair fibers from different body locations (Supplementary Fig. S2). Distinction of head, arm, and pubic hair is further enhanced by differential expression of intracellular proteins, which dominates the hair proteome^8^. Protein expression is much more similar between head and arm hair; notably, histone H3.1 exhibits higher expression in head and arm hair compared to pubic hair and is a key protein in differentiating these hair types (Supplementary Fig. S2). Found in the nucleosome, the protein is an integral component in chromatin structure and is linked to DNA synthesis and repair^38^, but also exhibits antimicrobial activity^28^. As histone H3 is localized to the cortex of hair shaft^39^, presence of variant H3.1 in head hair in high abundance is reasonable. Differentiation of hair fibers by body location based upon differential protein expression may be a valuable tool for screening in forensic investigations, but examination for any downstream effects on GVP and SNP identification is critically important for our forensic applications.

Effects of body location-specific proteomic variation on SNP identification were assessed by comparing protein subsets. Of the 37 differentially expressed proteins, 14% contain SNPs with variable exome-proteome consistency between sample replicates (Supplementary Fig. S3). These 8 SNPs comprise a minority, 12.3%, of all identified SNPs excluding false positives, and four SNPs show statistically significant positive correlations between protein abundance and frequency of response, including SNPs from K37. However, these SNPs are often not consistently identified among sample replicates, and in many cases, are not identified regardless of protein abundance, indicating that their absence is more likely attributed to a combination of the chemical characteristics of the GVP that suppresses their ionization and precursor ion competition for data-dependent MS/MS. Given the complexity of hair and that only the 10 most abundant ions per MS scan are fragmented, it is possible that the GVP is not sufficiently abundant to be selected for MS/MS in some samples. A key advantage of using data-dependent mass spectrometry is its breadth of proteome coverage, crucial in GVP discovery, but slight run-to-run differences in chromatographic separation may result in some irreproducibility in peptide selection for fragmentation^40,41^, contributing to unreliable SNP identification. Future development and operational use of targeted mass spectrometry approaches for GVP panels will eliminate unreliable SNP identification.

All exome-proteome consistent SNPs derive from hair structural proteins that do not exhibit body location-specific differential protein expression (Fig. 3), and no significant positive correlation exists between these SNPs and proteins (Supplementary Fig. S3). While SNPs from APOD, GSDMA, and K37 show some correlation with body location-specific protein abundance differences, there is no evidence to support universal SNP localization from body location-specific proteomic variation. For 97% of markers, SNP identification is independent of protein expression differences in head, arm, and pubic hair.

GVP candidates shown in Table 1 represent the peptides observed with the highest frequency in this set of single hairs. Notably, most GVPs are tryptic; however, many peptides from KAPs are non-tryptic. Instead, some of these peptides are cleaved at the C-terminal sides of cysteines or serines. Upon examination of their protein sequences, it is not surprising that non-tryptic GVPs are identified in KAPs, as there are few sites for trypsin to cleave among repeating chains of cysteine and serine near arginine-proline units. Because few tryptic sites exist, the frequency of identifying GVPs from KAPs is low compared to keratins^19^. Additionally, GVPs identified in structural proteins also contained missed cleavages, where the peptide contains one or more trypsin cleavage sites within its sequence. The presence of these peptides points to incomplete protein digestion by trypsin. Lower digestion efficiency in keratins is likely linked to their natural existence as coiled-coil structures through hydrophobic interactions^1^. Hair keratins are stabilized as coiled-coil heterodimers with surfactant^42^; removal of surfactant sodium dodecanoate during sample preparation likely resulted in the unfolding of hydrophobic proteins in a thermodynamically unfavorable environment, and subsequent protein aggregation^42^. Trypsin may have limited access to buried hydrophobic regions in keratins, thus reducing its digestion efficiency. However, a less efficient digestion can be beneficial when identifying unique peptides among these highly paralogous proteins^4,5^.

GVP markers from 8 robust SNPs yield highly consistent GVP profiles within an individual (Fig. 4 and Supplementary Fig. S4). Not surprisingly, the majority of GVPs derive from keratins and KAPs, though markers from keratins provide more discriminative power with respect to phenotype frequencies for RMP calculations. Greater discriminative power of GVPs from keratins arises from consistent identification of minor GVPs, as opposed to the more variable identification of peptide markers in KAPs for reasons discussed above. Sporadic GVP identification also contributes to sample replicate and within-individual variation, although, as expected, interindividual variation dominates GVP profile differences, ranging between 3 and 5 times that of intraindividual variation. The highly similar intraindividual GVP profiles and RMPs demonstrate not only robustness in identifying these particular GVP markers, but body location invariance with this protein-based strategy.

We determined equivalent probative value of head, arm, and pubic hair for protein-based human identification using genetically variant peptide markers in this study. Body location-specific proteomic variation was characterized, with GVP identification and SNP inference invariant across hair from different body locations. Further, a set of robust SNPs inferred from exome-proteome consistent GVPs yields similar differentiative potential of individuals from hair specimens irrespective of body location. While future development of targeted mass spectrometry methods for GVP identification will eliminate intraindividual variation, current methods successfully demonstrated body location-specific GVP invariance in single one-inch hairs, defined criteria for marker selection, and identified robust GVP markers for protein-based human identification.

## Materials and Methods

### Hair sample preparation for mass spectrometry

Head, arm, and pubic hair specimens from three subjects (ages 25, 31, and 35) were collected to profile the protein variation in non-chemically treated hair from different body locations, under approval by the Institutional Review Board at Lawrence Livermore National Laboratory (IRB ID# 15-008) and in accordance with the Common Rule. Written informed consent for specimen collection and analysis was obtained prior to collection. Samples were stored in the dark at room temperature (RT). A one-inch (25 mm) single hair was segmented from a hair sample, and each was further segmented into four pieces of equal length (~6 mm) for full immersion into the denaturation solution for protein extraction. To account for biological variation within individuals, different 25-mm single hair specimens from each body location were prepared as n = 4 for each individual. The same protocol was followed to prepare the first three sets of replicates for proteomics-only analysis; a fourth set of replicates was prepared with a slightly modified protocol for protein/DNA co-extraction.

Aliquots of 100 µL of an aqueous denaturation solution (50 mM dithiothreitol (DTT), 50 mM ammonium bicarbonate (ABC), and 20 mg/mL sodium dodecanoate (SDD)) were added to each single-inch hair specimen contained in a microcentrifuge tube. The tubes were sealed, placed in a 70 °C water bath, and ultrasonicated at a frequency of 37 MHz and 100% power (Elma, Singen, Germany) until each hair sample was entirely solubilized (on average, 2 h). 10 µL of 1 M iodoacetamide was added to each sample and the extracts were incubated in the dark at RT for 45 min.

To remove SDD (an ionization-suppressing compound in LC-MS/MS analysis) from the extracts, liquid-liquid extraction was performed using 100 µL of 0.75% (v/v) trifluoroacetic acid in ethyl acetate. The upper organic layer was removed after phase separation and each extract was re-adjusted to pH = 8 with 10 µL of 1 M ABC. Protein concentration was performed using spin filter concentrators with a lock volume of 20 µL (PES, 10 kDa MWCO; Thermo Fisher Scientific Inc., Waltham, MA). Samples were centrifuged at 3,000 × *g* for 15 min at RT, and 60 µL of buffer solution (50 mM DTT and 50 mM ABC) were added to wash the retentates, followed by a wash with 30 µL of buffer after centrifugation for 15 min. Finally, spin filter retentates were centrifuged for 30 min and reconstituted to 50 µL with buffer solution (50 mM DTT, 50 mM ABC, and 0.1 mg/mL ProteaseMAX™ Surfactant (Promega, Madison, WI)) prior to overnight trypsin digestion (TPCK-treated, sequencing grade) for at least 18 h accompanied by magnetic stirring at RT.

Protein digests were filtered for particulates using centrifugal filter tubes (PVDF, 0.1 µm; Millipore Sigma, Burlington, MA) with centrifugation at 9,000 × *g* for 10 min at RT. Filtered digests for the first three sets of replicates were then analyzed by LC-MS/MS. For the fourth set of replicates, a protein/DNA co-extraction procedure was performed. 200 µL of ethanol was added to each filtrate, and the mixture was transferred to a DNA-binding column from the QIAamp DNA Micro Kit (Qiagen, Hilden, Germany) and fractionated via centrifugation into a protein fraction (flow-through) and a DNA fraction (retentate). Protein digest eluate collected after centrifugation at 6,000 × *g* for 1 min at RT was evaporated to dryness under vacuum and reconstituted in 50 µL of 50 mM ABC, 50 mM DTT, and 0.1 mg/mL ProteaseMAX™ Surfactant. The reconstituted protein digest was filtered as above and analyzed via LC-MS/MS. Analysis and results from the DNA fraction will be described in a future publication.

### Liquid chromatography-tandem mass spectrometry analysis

Protein digests were analyzed on an EASY-nLC 1200 system coupled to a Q Exactive Plus Orbitrap mass spectrometer (Thermo Fisher Scientific Inc., Waltham, MA). 1 µL injections were loaded onto an Acclaim™ PepMap™ 100 C18 trap (75 µm × 20 mm, 3 µm particle size), washed, and separated on an Easy-Spray™ C18 analytical column (50 µm × 150 mm, 2 µm particle size). Separations were performed at a flow rate of 300 nL/min using mobile phases A (0.1% formic acid in water) and B (0.1% formic acid in 90% acetonitrile/10% water) over a 107-min gradient: 2 to 3% B in 1 min, 3 to 11% B in 75 min, 11 to 39% B in 15 min, ramped to 100% B in 1 min, and held at 100% B for 15 min. Positive mode nano-electrospray ionization was achieved at a voltage of 1.9 kV. Full MS scans were acquired at a resolution of 70,000, with a maximum ion accumulation time of 30 ms, and a scan range between *m/z* 380 and 1800. Data-dependent MS/MS scans were triggered for the 10 most abundant ions at an intensity threshold of 3.3 × 10^4^ and acquired at a resolution of 17,500, with a maximum ion accumulation time of 60 ms, dynamic exclusion of 24 s, and an isolation window of 2 Da. HCD fragmentation was performed at a collision energy setting of 27. Singly-charged species and ions with unassigned charge states were excluded from MS/MS.

### Protein and peptide identification

Mass spectral data were imported into PEAKS Studio 8.5 (Bioinformatics Solutions Inc., Waterloo, ON, Canada) for peptide identification via *de novo* sequencing and subsequent database searching. Precursor ion mass tolerance was selected as ± 20 ppm, while a mass error of 0.05 Da was allowed for fragment ions. A list of 313 potential post-translational modifications, which includes cysteine carbamidomethylation, methionine oxidation, and asparagine and glutamine deamidation, was allowed as variable modifications for peptide identification. The maximum number of PTMs allowed per peptide was three, and 3 tryptic missed cleavages on either end of the peptide were permitted. All *de novo*-sequenced peptides with a confidence score (−10l gP) greater than 15% were matched to protein sequences in a reference database. To capture non-mutated proteins in the samples, the UniProtKB SwissProt Human protein database (downloaded September 27, 2017) was used for protein inference from identified peptides^43^. A second protein and peptide identification using the same raw mass spectral files was performed in PEAKS, where *de novo*-sequenced peptides were searched against individualized protein databases created from exome sequence data (Supplementary Methods). The second PEAKS analysis enabled a focused search for proteins with expected mutations to identify GVPs in each sample. Each individualized protein database contains protein sequences from a list of 691 common gene products found in hair with the appropriate mutations expected in an individual based on their exome sequence. GVPs identified from each hair specimen were matched to mutated protein sequences in individualized protein databases in the second peptide identification analysis.

Identified proteins and peptides were further filtered with a 1% false-discovery rate threshold for peptide-spectrum matches and then exported from PEAKS. An in-house Python-based script was applied to the output files to merge results from the two PEAKS analyses and generate a non-redundant protein profile for each sample. Protein profile metrics include the number of proteins, unique peptide sequences, amino acids, and SNPs identified from both major and minor GVPs.

### GVP profile generation – observed phenotype frequencies

Each GVP profile was established using the presence or absence of the major and minor GVPs at each SNP locus. Observed phenotype frequencies were used to represent the presence or absence of GVPs. Conventionally, SNPs are associated with population genotype frequencies, obtained from the Genome Aggregation Database (gnomAD)^44^. However, to account for uncertainty in establishing a genotype with proteomic responses from incomplete GVP detection, observed phenotype frequencies were used as sums of genotype frequencies (i.e., sum of either major homozygote or minor homozygote genotype frequency with heterozygote genotype frequency) when only either a major or minor GVP was detected. Total population genotype frequencies from gnomAD were used. For example, detection of only the minor GVP for a SNP resulted in an observed phenotype frequency as the sum of the heterozygote and minor homozygote frequencies. Observed phenotype frequency was not reported for absent GVPs (i.e., one true negative and one false negative response) as the SNP was not considered in that sample.

### Statistical analysis

All statistical comparisons were performed in R (x64 version 3.4.4). Significance was established at α = 0.05. Two-way ANOVAs were performed using the *aov* function after fitting a linear model in the *stats v3.5.0* package. Tukey HSD post-hoc tests, two sample t-tests, and tests for association of Pearson product-moment correlations were performed using the same package. Equal variances were not assumed for t-tests. For non-parametric Kruskal-Wallis and Dunn post-hoc tests, the *agricolae v.1.2*–*8* package and *dunn.test v.1.1.0* package, respectively, were used, and a Bonferroni correction was applied to adjust p-values. Principal components analysis (PCA) was performed in MATLAB (R2017a, MathWorks, Natick, MA). All plots were drawn in OriginPro 2018 (OriginLab Corp., Northampton, MA) except for MATLAB outputs; PCA plots were drawn in Microsoft Excel 2016 (Redmond, WA). All values are reported as m ± s.d. unless otherwise specified.

### Disclaimer

The Lawrence Livermore National Laboratory, Office of Scientific and Technical Information, Information Management (IM) number is: LLNL-JRNL-757511. This document was prepared as an account of work sponsored by an agency of the United States government. Neither the United States government nor Lawrence Livermore National Security, LLC, nor any of their employees makes any warranty, expressed or implied, or assumes any legal liability or responsibility for the accuracy, completeness, or usefulness of any information, apparatus, product, or process disclosed, or represents that its use would not infringe privately owned rights. Reference herein to any specific commercial product, process, or service by trade name, trademark, manufacturer, or otherwise does not necessarily constitute or imply its endorsement, recommendation, or favoring by the United States government or Lawrence Livermore National Security, LLC. The views and opinions of authors expressed herein do not necessarily state or reflect those of the United States government or Lawrence Livermore National Security, LLC, and shall not be used for advertising or product endorsement purposes.

## Supplementary information

Supplementary Information

Supplementary Table

## Data Availability

The mass spectrometry proteomics data generated during the current study have been deposited to the ProteomeXchange Consortium (http://proteomecentral.proteomexchange.org) via PRIDE with identifier PXD010982.
