# Supplementary material for: Hair Proteome Variation at Different Body Locations on Genetically Variant Peptide Detection for Protein-Based Human Identification
Source: Sci Rep. 2019 May 21;9:7641. doi: 10.1038/s41598-019-44007-7 (PMC6529471; doi:10.1038/s41598-019-44007-7)
Supplement: Supplementary file 1 — Supplementary Information [file 41598_2019_44007_MOESM1_ESM.pdf]

Hair Proteome Variation at Different Body Locations on Genetically Variant Peptide Detection for Protein-Based Human Identification

Fanny Chu[1,2], Katelyn E. Mason[1], Deon S. Anex[1]*, A. Daniel Jones[2,3], Bradley R. Hart[1]

[1]Forensic Science Center, Lawrence Livermore National Laboratory, 7000 East Ave., Livermore, CA 94550
[2]Department of Chemistry, Michigan State University, 578 S Shaw Ln, East Lansing, MI 48824
[3]Department of Biochemistry and Molecular Biology, Michigan State University, 603 Wilson Rd, East Lansing, MI 48824

***Corresponding Author:**
Deon S. Anex
anex1@llnl.gov

Supplementary Methods

Exome Sequencing and Genetically Variant Peptide Prediction

A detailed description of this process can be found elsewhere[1]. Briefly, full exome sequencing was performed using DNA isolated from blood samples of individuals who provided a hair sample (ACE Research Exome with Secondary Analysis; Personalis Inc., Menlo Park, CA). Variant call format files from exome sequencing were then filtered to include only missense variants (SNPs) from 691 genes commonly found in proteomic analyses. Sequence data quality of PASS or better (according to scoring system by the Genome Analysis Toolkit (Broad Institute)) was applied to filter the subset of SNPs. Conversion of sequence data to the current Genome Reference Consortium Human Build 38 (GRCh38) from GRCh37.5 coordinates was performed using the Bioconductor package Variant Annotation in R. Variant lists were further annotated using ENSEMBL's Variant Effects Predictor to include transcript, genetic mutation location, and corresponding amino acid substitution for each SNP. Human Genome Variation Society (HGVS) notation was used to specify SNPs in the absence of Reference SNP IDs. ENSEMBL Genome Browser transcripts associated with the subset of SNPs were downloaded using the R package BiomaRt and altered to reflect the genetic mutation. Original and altered transcripts were then used to create protein sequences, both with and without amino acid variants, in R. Mutated and their non-mutated counterpart protein sequences were combined and converted into FASTA files to be used as individualized protein databases for each subject.

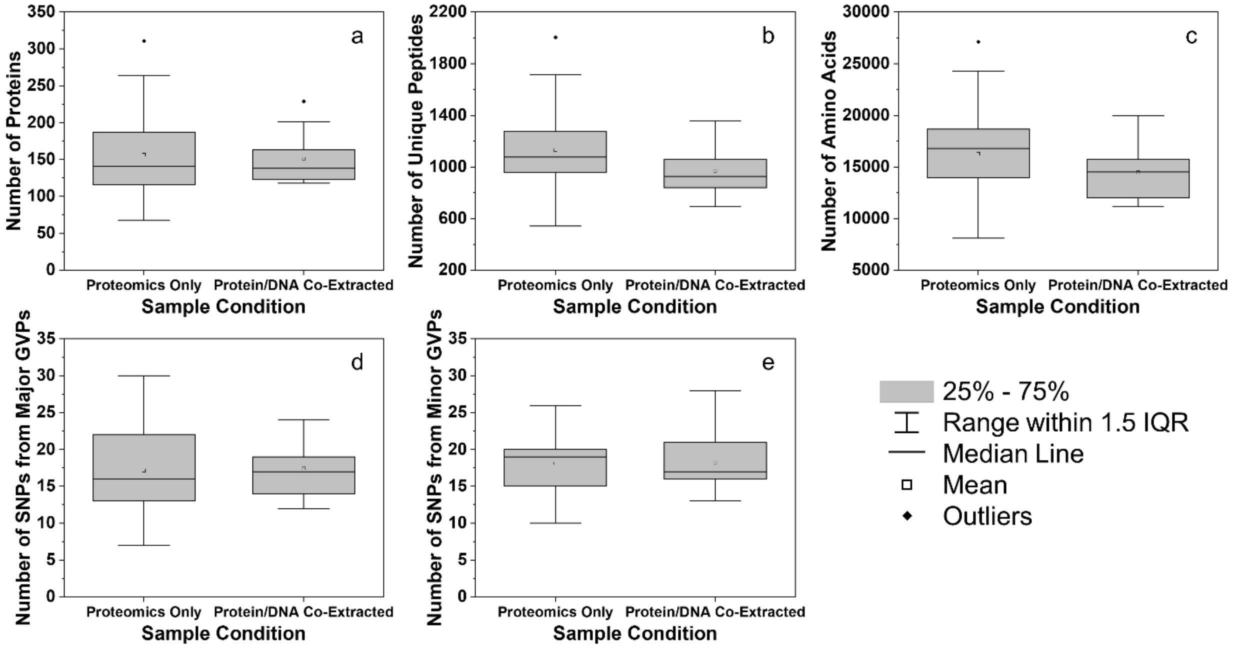

Supplementary Figure S1. Comparison of average numbers of identified (a) proteins, (b) unique peptides, (c) amino acids, and missense SNPs inferred from (d) major and (e) minor GVPs between Proteomics Only (n = 27) and Co-Extracted (n = 9) samples. Protein/DNA Co-Extracted samples are not statistically different from Proteomics Only samples (two sample t-test; $p \geq 0.106$).

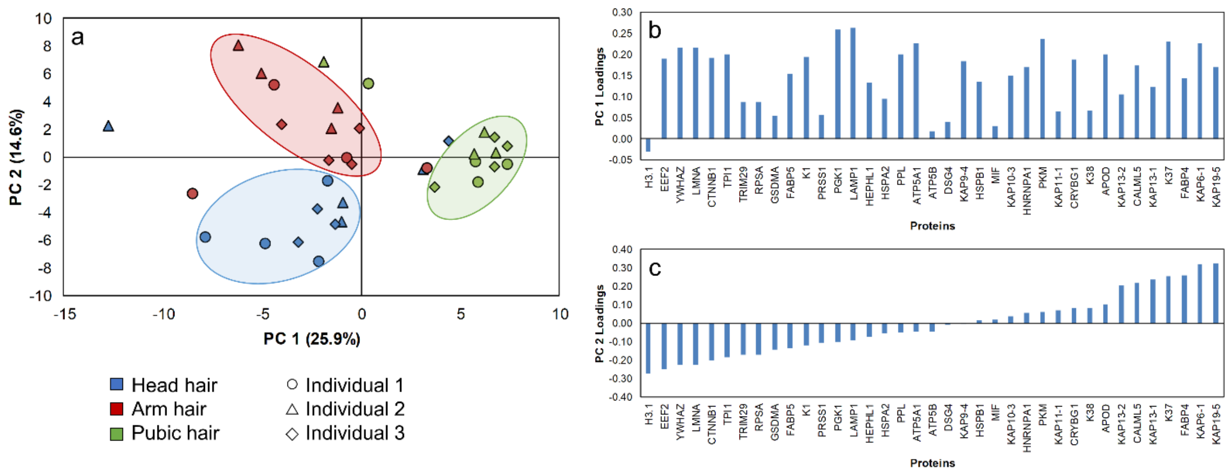

Supplementary Figure S2. Principal components analysis (a) scores and (b), (c) PCs 1 and 2 loadings using 37 differentially expressed hair proteins. Protein abundances were log-transformed and Pareto-scaled to reduce bias from highly abundant proteins. This subset of proteins allows distinction of hair from different body locations; 75% of samples are captured in non-overlapping clusters.

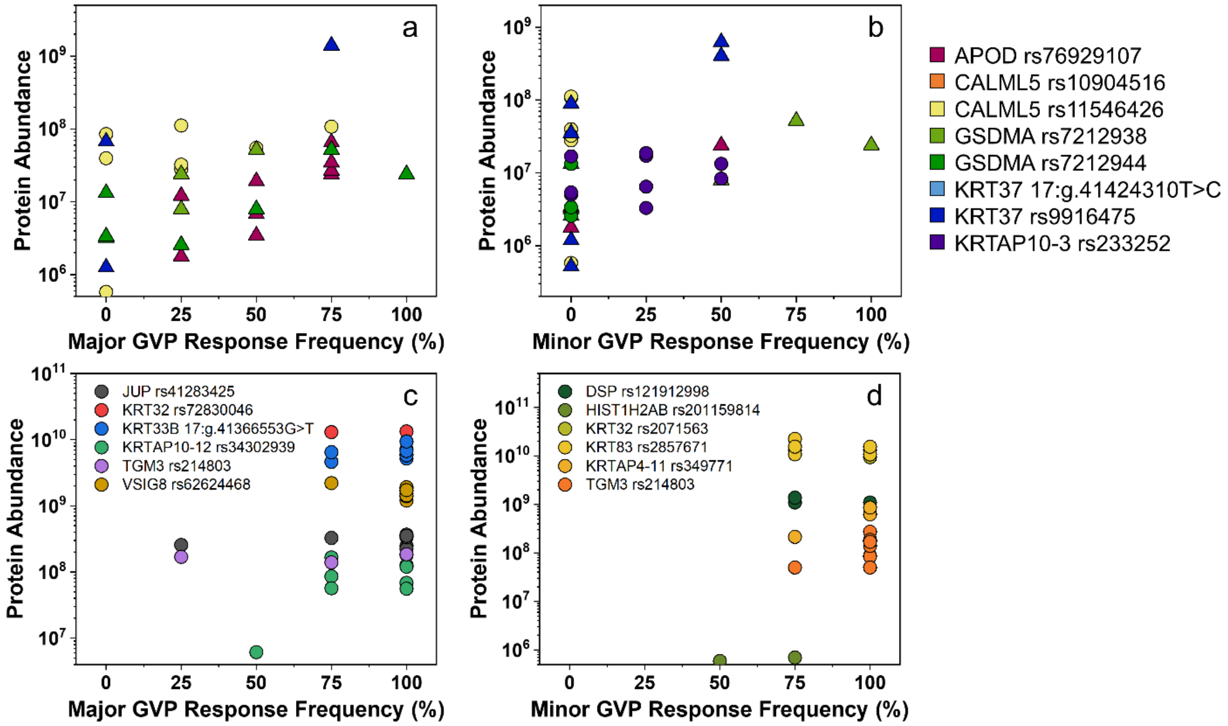

Supplementary Figure S3. Correlations between GVP response frequency and abundances of differentially expressed proteins for SNPs identified from (a) major GVPs and (b) minor GVPs. Identified SNPs in (a) and (b) are not exome-proteome consistent and display variation in sample replicates. (c) and (d) illustrate the relationship between GVP response frequency of unreliably identified exome-proteome consistent SNPs and protein abundance. Triangles denote significant positive correlations between GVP response frequency for a SNP and corresponding protein abundance (Pearson product-moment correlation; $n = 9$; $p \leq 0.043$). GVP responses show some correlation with protein abundance for SNPs in APOD, GSDMA, and KRT37, but the majority of GVP identification is not affected by differential protein expression.

Gene	SNP Identifier	1-H.1	1-H.2	1-H.3	1-H.4	1-A.1	1-A.2	1-A.3	1-A.4	1-P.1	1-P.2	1-P.3	1-P.4
KRT33A	rs148752041	1	1	1	1	1	1	1	1	1	1	1	1
VSIG8	rs62624468	0	0	0	0	0	0	0	0	0	0	0	0
KRT81	rs2071588	1	1	1	1	1	1	1	1	1	1	1	1
KRT83	rs2852464	0,1	0,1	0,1	0,1	0,1	0,1	0,1	0,1	0,1	0,1	0,1	0,1
KRT32	rs2071563	0	0	0	0	0	0	0	0	0	0	0	0
KRTAP10-9	rs9980129	--	1	--	--	--	--	--	--	--	1	1	--
KRTAP10-3	rs233252	--	--	--	--	--	--	--	--	--	--	--	--
FAM83H	rs9969600	--	--	--	--	--	--	--	--	--	--	--	--

Gene	SNP Identifier	2-H.1	2-H.2	2-H.3	2-H.4	2-A.1	2-A.2	2-A.3	2-A.4	2-P.1	2-P.2	2-P.3	2-P.4
KRT33A	rs148752041	--	--	--	--	--	--	--	--	--	--	--	--
VSIG8	rs62624468	0,1	0,1	0,1	0,1	0,1	0,1	0,1	0,1	1	0,1	0,1	0,1
KRT81	rs2071588	--	--	--	--	--	--	--	--	--	--	--	--
KRT83	rs2852464	0,1	0,1	0,1	0,1	0,1	0,1	0,1	0,1	0,1	0,1	0,1	0,1
KRT32	rs2071563	0,1	0	0,1	0,1	0,1	0,1	0,1	0,1	0,1	0,1	0,1	0,1
KRTAP10-9	rs9980129	1	1	1	--	--	--	1	--	--	1	1	--
KRTAP10-3	rs233252	--	--	1	--	--	--	--	1	--	1	1	--
FAM83H	rs9969600	1	--	--	--	--	--	--	--	1	--	--	--

Gene	SNP Identifier	3-H.1	3-H.2	3-H.3	3-H.4	3-A.1	3-A.2	3-A.3	3-A.4	3-P.1	3-P.2	3-P.3	3-P.4
KRT33A	rs148752041	--	--	--	--	0	--	--	--	--	--	--	--
VSIG8	rs62624468	0	0	0	0	0	0	0	0	0	0	0	0
KRT81	rs2071588	1	1	1	1	1	1	1	1	1	1	1	1
KRT83	rs2852464	0	0	0	0	0	0	0	0	0	0	0	0
KRT32	rs2071563	0	0	0	0	0	0	0	0	0	0	0	0
KRTAP10-9	rs9980129	--	1	1	--	--	--	1	--	--	1	1	--
KRTAP10-3	rs233252	1	--	1	--	1	--	--	--	--	--	1	--
FAM83H	rs9969600	--	--	--	--	--	--	--	--	--	--	--	--

Supplementary Figure S4. GVP profiles established for each sample using the presence or absence of major and minor GVPs. “0” and “1” represent the presence of the major and minor GVP, respectively, while “--” represents the absence of GVPs.

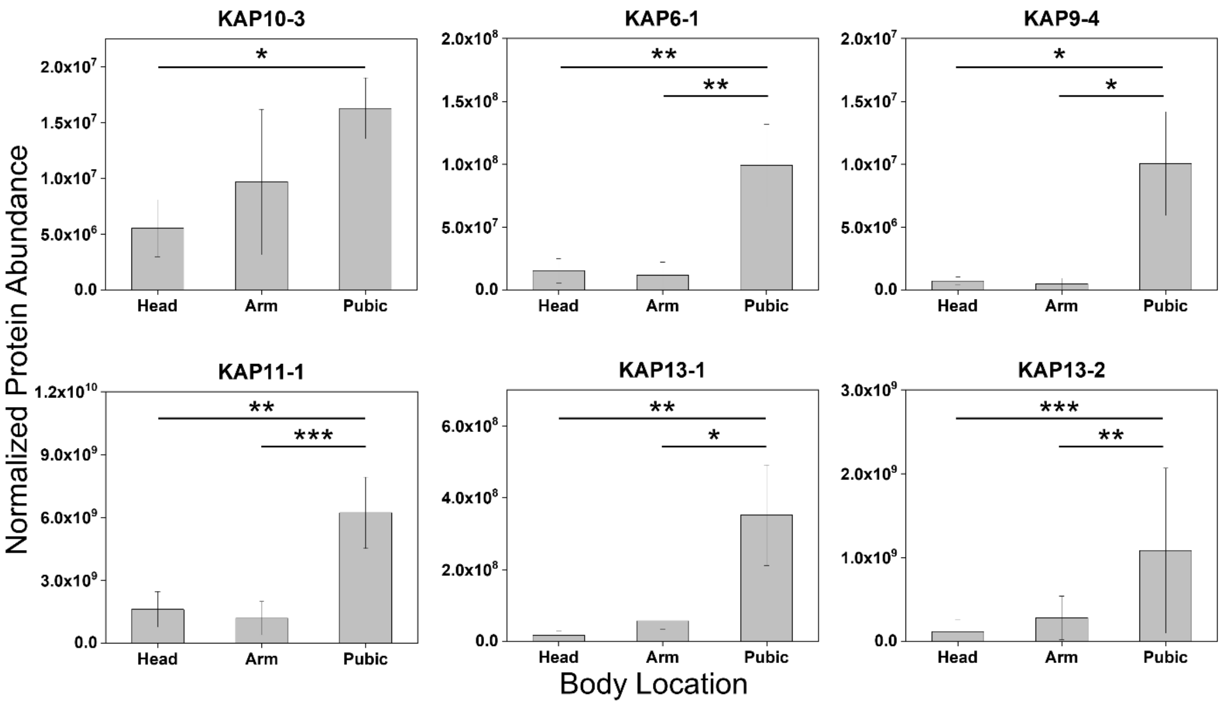

Supplementary Figure S5. Average abundances for a subset of differentially expressed hair proteins at different body locations (two-way ANOVA and Tukey HSD; n = 36). Error bars represent standard deviation from 4 replicate measurements of each of three individuals. Black lines represent statistically significant comparisons and significance levels are represented as: $p \leq 0.05$ (*), $p \leq 0.01$ (**), and $p \leq 0.001$ (***).

Reference

1 Mason, K. E., Paul, P. H., Chu, F., Anex, D. S. & Hart, B. R. Development of a Protein-based Human Identification Capability from a Single Hair. *Journal of Forensic Sciences* **0**, doi:10.1111/1556-4029.13995 (2019).
